# Language-Based Modulation of the Stream/Bounce Judgment

**DOI:** 10.1177/2041669520935925

**Published:** 2020-06-26

**Authors:** Shengbin Cui, Atsunori Ariga

**Affiliations:** Graduate School of Humanities and Social Sciences, Hiroshima University

**Keywords:** stream/bounce perception, emotional processing, response bias

## Abstract

When two identical objects on a screen move toward each other, coincide at the center of the screen, and then continue to move along their original trajectories to the opposite starting points, observers perceive these visual stimuli as showing one of the two possible scenarios: streaming through or bouncing off each other (stream/bounce perception). Previous research has shown that when a high-arousal face is presented along with the two moving objects, the bouncing percept was predominant, as compared with when a middle- or low-arousal face is presented. In this study, however, such a modulatory effect of the emotional face was eliminated when participants did not judge stream or bounce and the terms “bouncing/streaming” were not used in the experiments. These results suggest that the modulatory effect of emotional stimuli on the stream/bounce judgment cannot be explained solely by the emotional processing per se but, rather, can be modulated by language-based processing.

When two identical objects on a screen move toward each other, coincide at the center of the screen, and then continue to move along their original trajectories to the starting positions of the other object, observers perceive these visual stimuli as one of the two situations: (a) streaming through or (b) bouncing off each other (stream/bounce perception, Metzger, 1934). Focusing on this stream/bounce bistable perception, previous research has investigated how the visual system establishes perceptual representations for ambiguous motion stimuli. For example, when a sound (or an oddball sound) is presented simultaneously with the coincidence of the two objects, observers’ perceptions are biased toward the bouncing percept (Sekuler et al., 1997; Watanabe & Shimojo, 2001). The neural mechanism responsible for this audiovisual bounce-inducing effect (ABE) may be related to the activity in the right posterior parietal cortex, which is thought to be responsible for attention and binding of cross-modal information (Maniglia et al., 2012). Importantly, Grassi and Casco (2010) argued that early sensory and higher perceptual (or decisional) processes are both responsible for the ABE, and that lower order processes may be modulated by higher order auditory information. Given these studies, converging evidence suggests that stream/bounce perception is affected by both lower order and higher order processes.

In fact, Gobara et al. (2018) have demonstrated the effect of the higher order processing (i.e., emotional processing) on the stream/bounce perception. As emotional visual stimuli, emoticons, which are face images composed of numbers, letters, or punctuation marks, were used to investigate their effects on the stream/bounce perception. When a high-arousal emoticon (e.g., ‘(°Д°)’) was added to the motion display, participants predominantly reported the bouncing percepts for two moving objects compared with when a middle- (e.g., ‘(‘ω’)’) or low-arousal (e.g., ‘(˙-˙)’) emoticon was added. Their results suggest that higher order emotional processing influences the bistable perception of two interactive objects.

That said, we believe that their interpretation of the results, as the modulatory effects of emotional processing on “perception” of stream/bounce, is premature. Because they asked participants to judge whether they perceived the two objects as streaming or bouncing after every trial, they did not completely rule out the artificial effects of a language-emoticon association on the stream/bounce judgment. In other words, participants were likely to choose a bouncing response, irrespective of their stream/bounce perception, upon the high-arousal emoticon just following the association between the high-arousal term “bouncing” and emoticon ‘(°Д°)’ resulting in more reports of bouncing for the high-arousal display compared with the middle- or low-arousal displays.

Indeed, Gobara et al. investigated such a response bias in Experiment 5 of their study by manipulating when the emoticon appeared on the display. As the presentation of emoticons after the coincidence of two moving objects had no effect on the stream/bounce judgment, their results were concluded as reflective of the stream/bounce “perception,” not the response bias. However, we believe Experiment 5 to be insufficient to exclude the response bias hypothesis, since the bias, if any, is supposed to be maximal when the emoticon appears just at the coincidence of two objects. Because the participants become extremely conscious of (or attentive to) stream/bounce at the moment of coincidence, compared with other times, the chance of linguistically associating the stream/bounce representation with the emoticon increases when it appears exactly at the same time as the coincidence. Therefore, in this study, we investigated whether language-based processing modulates the stream/bounce judgment in a strict manner, in addition to replicating the findings reported by Gobara et al. (2018). More specifically, to exclude language-based response bias completely, we never used the term “bouncing/streaming” or the task that requires stream/bounce judgment in the stream/bounce experiment.

## Experiment 1

### Method

#### Ethics Statement

All experimental procedures were reviewed and approved by the Institutional Review Board of Hiroshima University, Japan. Written informed consent was obtained from all participants both before and after the experiment.

#### Participants

A total of 12 Japanese students (3 males and 9 females; mean age = 20.5 years) who were naive to the purpose of this study participated in this experiment. They had normal or corrected-to-normal vision. This sample size was determined according to the method of Gobara et al. (2018).

#### Stimuli

All stimuli used in this study were identical to those used in Experiment 2 in Gobara et al. (2018), which reported the significant effect of arousal of emoticons on the stream/bounce judgment. The objects used were two black discs (1° in visual angle in diameter) presented on a gray square background (14.20° × 14.20°, 49.67 cd/m^2^). The center of the gray square background equaled that of the monitor. The starting positions of the discs were set to the left and right of the center of the gray square background. The distance between the two discs was 12.20°. They moved toward each other with a uniform rectilinear motion of 6.21 deg/s, coinciding at the center of the gray square background and continuing to move to the opposite starting points along the virtual horizontal line. After reaching the ends of their trajectories, the objects stopped moving and disappeared. The entire duration of this motion display was approximately 1,990 milliseconds.

**Figure 1.**
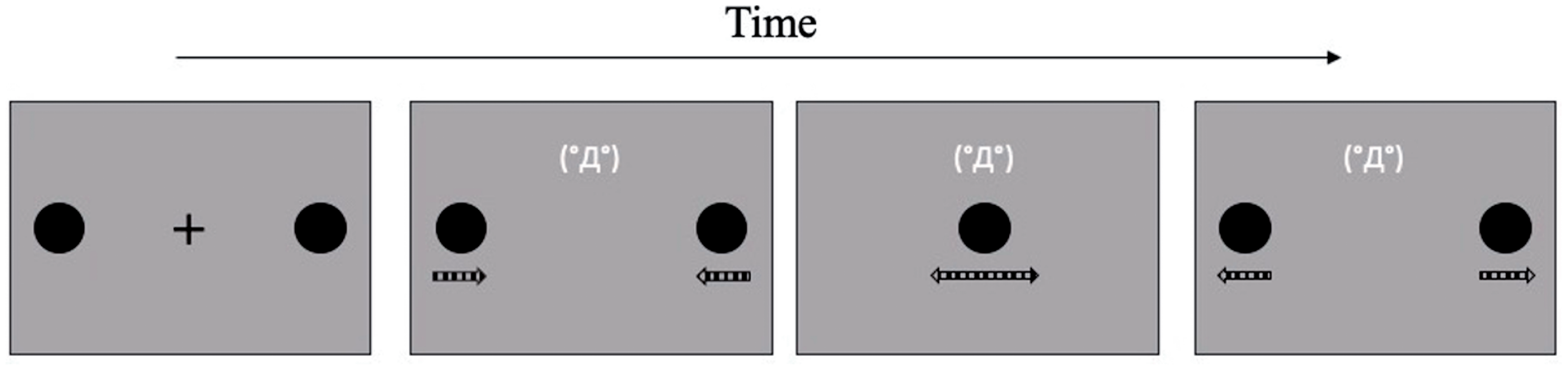
An Example of the Stream/Bounce Display With a High-Arousal Emoticon in Experiment 1. The dashed arrows below the discs, which were not presented in the actual experiment, indicate the motion directions of two black discs.

In this study, three emoticons were used: (°Д°), (‘ω’), and (˙-˙), which were representative of high-, middle- and low-arousal emotional stimuli, respectively. These were presented in the font of Hiragino Kaku Gothic Pro, with a size of 25 pt. They were shown in white color and displayed 2.38° above the center of the gray square background. The emoticon appeared while the black discs were moving (Figure 1). The distance between the monitor and the participants was 57 cm.

#### Procedure

The procedure of Experiment 1 was identical to that of Experiment 2 in Gobara et al. (2018). The experiment was conducted with each participant individually in a dark room. The participants started each trial by pressing the space bar. Then, the black fixation cross was presented at the center of the gray square background for 1,000 milliseconds. The two black discs were displayed 500 milliseconds after the appearance of the fixation cross. After another 500 milliseconds, the fixation cross disappeared, and one of the emoticons appeared when the discs started moving toward each other. When they reached the ends of their set trajectories, the discs and the emoticon disappeared. Following the display, participants reported whether they perceived streaming or bouncing for two objects by pressing ‘J’ for the streaming percept and ‘F’ for the bouncing percept on the keyboard. The experimental trials consisted of three emoticons (high-, middle-, and low-arousal) × 20 repetitions, a total of 60 trials, which was preceded by 5 practice trials. The order of the emoticons presented in the trials was random.

### Results

**Figure 2.**
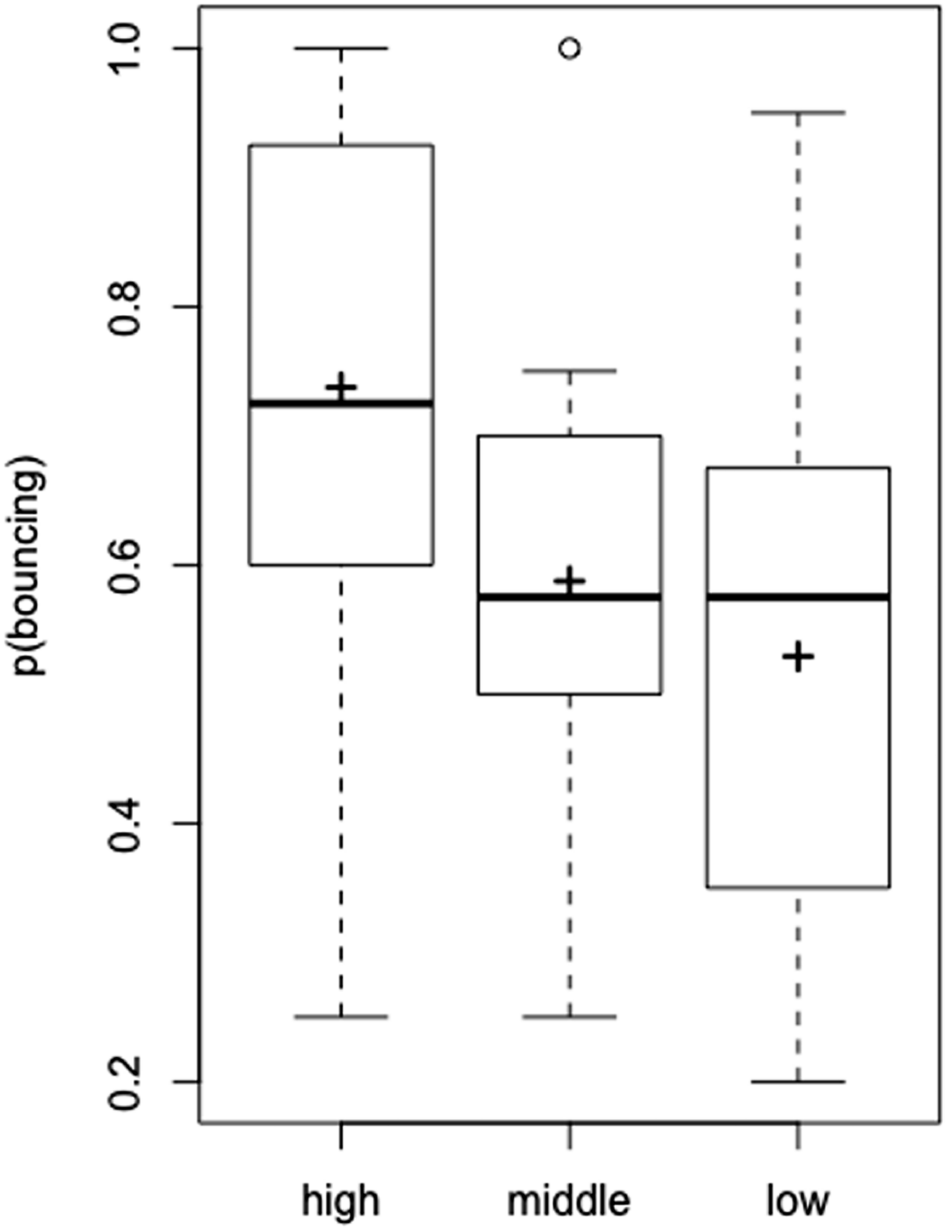
The Proportion of Bouncing Responses in Experiment 1. The black cross on the boxplot represents the average proportion of participants perceiving bouncing.

The proportion of the bouncing response by each participant was set as the dependent variable. The mean proportion of the bouncing response is shown in Figure 2. A within-subject one-way analyses of variance (ANOVAs) demonstrated a significant main effect of the emoticon, *F*(2, 22) = 8.144, *p* = .002, partial η^2^ = 0.425. Multiple comparisons showed that the proportion of the “bouncing” response in the high-arousal condition was significantly higher than that in the other conditions (*p* < .05).

Based on post hoc power analyses using G*Power (Faul et al., 2007), a sample size of 12 with the present factorial design, a significance level of 0.05, and the effect size observed in this experiment (partial η^2^ = 0.425) yielded a 99.99% chance of correctly rejecting the null hypothesis.

### Discussion

We replicated the previously reported phenomenon by Gobara et al. (2018). Therefore, the phenomenon of a high-arousal stimulus boosting the bouncing response compared with the middle- and low-arousal stimuli is robust and replicable.

It is noteworthy that the bouncing response was overall predominant under all conditions, compared with the typical stream/bounce situations (e.g., Grassi & Casco, 2012; Watanabe & Shimojo, 1998, 2001), which includes 10% to 20% reports of bouncing. Because the mere presence of a face stimulus (or an emoticon in our study) exogenously captures participants’ attention, even though it is task irrelevant (e.g., Ariga & Arihara, 2018), such an inattention situation may have biased the participants’ responses toward bouncing in this study, just like ABE (Watanabe & Shimojo, 1998, 2001).

Next, we investigated whether this arousal effect persists even though the language-based response bias is omitted.

## Experiment 2

### Method

#### Participants

A total of 12 Japanese students (5 males and 7 females; mean age = 19.3 years) who were naive to the purpose of this study participated in this experiment. All had normal or corrected-to-normal vision. This sample size was the same as that in Experiment 1.

#### Stimuli and Procedure

**Figure 3.**
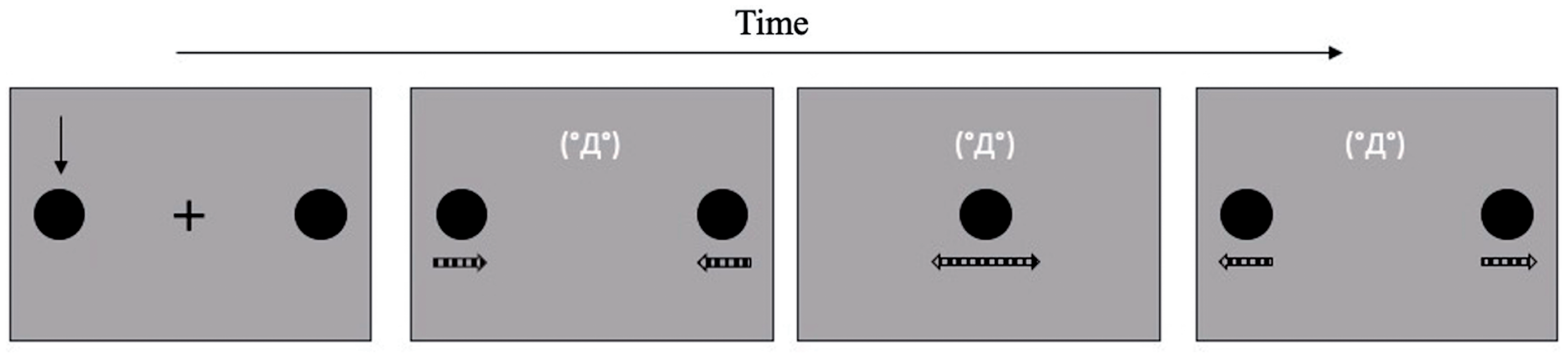
An Example of the Stream/Bounce Display With a High-Arousal Emoticon in Experiment 2. The dashed arrows below the discs, which were not presented in the actual experiment, indicate the motion directions of the two black discs.

The stimuli and procedure were identical to those used in Experiment 1, except for the following changes. When the black discs appeared in the display, a black arrow sign (Arial font, 40 pt. size) was presented earlier either the right or left disk for 500 milliseconds; the arrow disappeared when the discs started to move (Figure 3). Participants were required to report the final position of the disc (target) initially indicated by the arrow. If participants perceived that the target disc ended up at the left side, they pressed the ‘6’ key on the keyboard; if they perceived that the target ended up at the right side, they pressed the ‘space’ key. Participants performed 60 experimental trials in total. Importantly, we never used the term “bouncing/streaming” during the experiment and the task never required the stream/bounce judgment.

### Results

**Figure 4.**
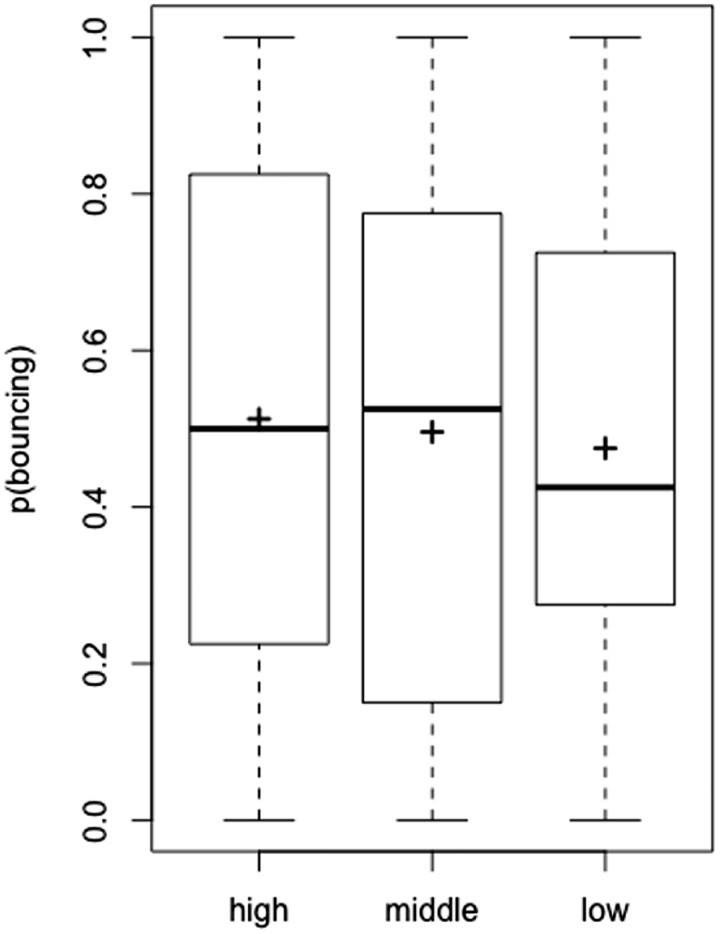
The Proportion of Bouncing Responses in Experiment 2. The black cross on the boxplot represents the average proportion of participants who perceived bouncing.

When the participants reported that the target disc returned to the starting position, we determined that the bouncing percept had occurred. On the other hand, when participants reported that the target disc reached at the opposite position, we determined that the streaming percept had occurred. The proportion of trials in which the participants perceived bouncing was used as the dependent variable, as in Experiment 1 (Figure 4). A within-subject one-way ANOVA showed no significant main effect of the emoticon, *F*(2, 22) = 0.965, *p* = .396, partial η^2^ = 0.081.

### Discussion

When the term “bouncing/streaming” was not used during the experiment and the task did not require the stream/bounce judgment, the arousal conveyed by the emoticon did not affect the stream/bounce perception. Apparently, the absence of the explicit use of the arousing word (i.e., “bouncing”) attenuated the effect, indicating the potential role of language-based processing in Experiment 1 and Gobara et al. (2018). These results can be interpreted in terms of our idea, that previous participants were likely to choose the bouncing response when the high-arousal emoticon was used, due to the association between the high-arousal term “bouncing” and emoticon.

Previous research (e.g., Watanabe & Shimojo, 1998, 2001) suggests that inattention situations generally bias participants’ responses toward bouncing. However, this was not the case in this experiment in which attention was not directed to the stream/bounce event. This discrepancy might be due to the difference between our and their methods. In their experiments, the transient event (or distractor) was additionally presented around the timing of the target superimposition, whereas no transient event occurred in our experiment, and thus the participants pursued one target from the beginning of the trial. That is, the focus of attention (distractor vs. target) may have elicited the difference between the results of our and their studies.

However, the task in this experiment differed somewhat to that in Experiment 1 and in Gobara et al. (2018). In this experiment, participants observed the stimulus display and paid attention to only the target disc, whereas previous participants were likely to divide their attention between the two discs, which may suppress the bouncing percept and make the arousal effect invisible. Therefore, the next experiment investigated the stream/bounce perception under the circumstance in which the task did not require the stream/bounce judgment, as in Experiment 2, but did require split attention, as in Experiment 1 and Gobara et al.

## Experiment 3

### Method

#### Participants

A total of 12 Japanese students (3 males and 9 females; mean age = 19.8 years) participated in this experiment. All had normal or corrected-to-normal vision and all were naive to the purpose of this study. The sample size was the same as that in the previous experiments.

#### Stimuli and Procedure

**Figure 5.**
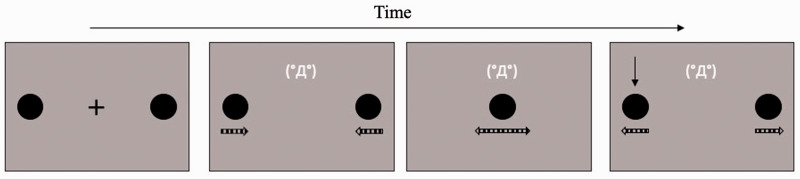
An Example of the Stream/Bounce Display With the High-Arousal Emoticon in Experiment 3. The dashed arrows below the discs, which were not presented in the actual experiment, indicate the directions of the motion of the two black discs.

The stimuli and procedure were identical to those used in Experiment 2, except for the following changes. The arrow sign that indicates target did not appear at the beginning of the trial, but, rather, it appeared at the end, when the motions of two discs had finished. After the 500 milliseconds, the arrow and two discs disappeared (Figure 5). The participants were required to report the initial position of the disc indicated by the arrow sign. If the participants perceived the target disc to have come from the left, they pressed the ‘6’ key on the keyboard; if they perceived the target to have come from the right, they pressed the ‘space’ key. The participants performed 60 experimental trials in total. Note that, again, we never used the term bouncing/streaming during the experiment and the task never required the stream/bounce judgment.

### Results

**Figure 6.**
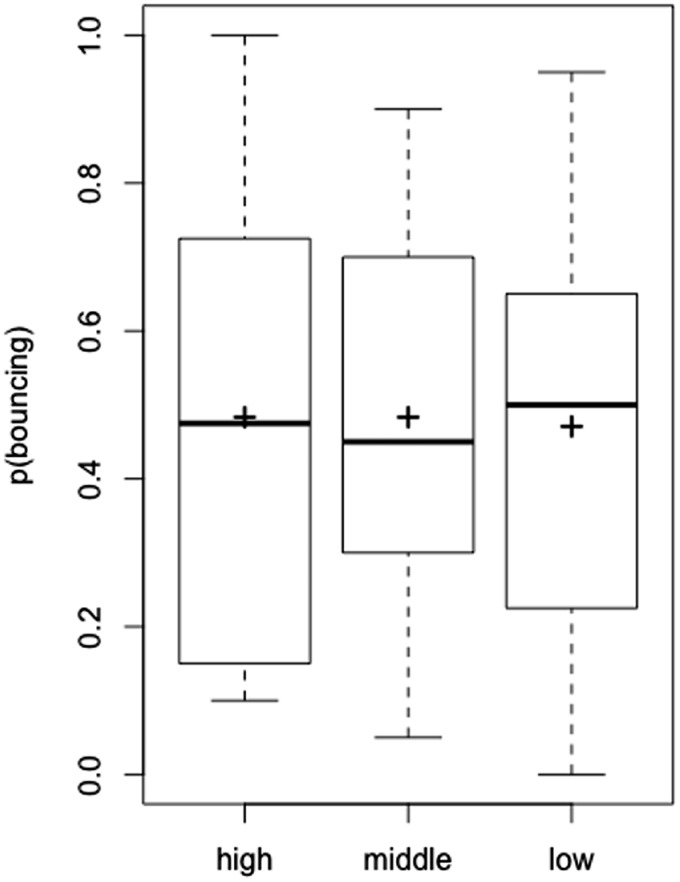
The Proportion of Bouncing Responses in Experiment 3. The black cross on the boxplot represents the average proportion of participants who perceived bouncing.

When the participants reported that the target disc came from the same position, we determined that the bouncing percept had occurred. On the other hand, when the participants reported that the target disc came from the opposite side, we determined that the streaming percept had occurred. The proportion of trials in which the participants perceived bouncing was set as the dependent variable, as in Experiments 1 and 2 (Figure 6). A within-subject one-way ANOVA showed no significant main effect of the emoticon, *F*(2, 22) = 0.090, *p* = .914, partial η^2^ = 0.008.

### Discussion

Again, the emotional stimuli did not affect the stream/bounce perception when the terms “bouncing/streaming” and the stream/bounce judgment task were not used. The results of Experiment 2 were replicated even though the task required the participants to split their attention between two discs, as in Experiment 1 and Gobara et al. (2018). Therefore, we conclude that the presence of the high-arousal emoticon ‘(°Д°)’ alone is not sufficient to elicit the reliable arousal effect observed in Experiment 1 and Gobara et al., but rather it elicits this effect only in conjunction with the high-arousal term “bouncing.”

## General Discussion

This study aimed to investigate how language processing contributes to the stream/bounce judgment. We focused on the phenomenon reported previously by Gobara et al. (2018), namely, that the high-arousal emoticon boosts the bouncing response compared with the middle- and low-arousal emoticons. Because the previous study did not completely rule out the response bias, the possibility that the association between the high-arousal term “bouncing” and emoticon ‘(°Д°)’ (i.e., the language-based response bias), and not the emotional processing per se, could explain the phenomenon remained. This study addressed this issue and investigated the robustness of the phenomenon (Gobara et al., 2018).

In Experiment 1, we succeeded in replicating the arousal effect in the stream/bounce judgment. That is, the bouncing response was facilitated when the high-arousal emoticon was presented with the moving objects. In Experiments 2 and 3, we did not use the term “bouncing/streaming” during the experiment and the stream/bounce judgment task; instead, we required the participants to report the initial or final positions of a target disc. If the language-based processing had nothing to do with the arousal effect observed in Experiment 1 and Gobara et al. (2018), the effect of the emoticon should have been observed under these circumstances; but this was not the case. That is, the arousal effect in the stream/bounce judgment was attenuated when the term “bouncing/streaming” and the stream/bounce judgment task were not explicitly used. Taken together, we can conclude that the bouncing percept is unlikely to be established by emotional processing itself, and that the bouncing response is likely to be selected by the language-based response bias (i.e., the associated “bouncing” emoticon). To support this idea, we conducted a supplementary experiment (*N* = 12) to test whether the term “bouncing” is indeed high arousal compared with the term “streaming.” Twelve Japanese students (10 males and 2 females; mean age = 22.0 years) rated how arousing each term was on a 7-point Likert-type scale (1 = *low arousal*, 7 = *high arousal*). The rating score for the arousal level was significantly higher for the term “bouncing” than for the term “streaming,” paired-sample *t*(11) = 7.532, *p* < .001, *d_z_* = 2.174. Therefore, the term “bouncing” is relatively high arousal, and we can associate it with the high-arousal emoticon.

That said, several explanations are also possible regarding the arousal effect observed in Experiment 1 presented here and in Gobara et al. (2018) at various stages of processing. First, the associated “bouncing/streaming” emoticon may affect the percepts themselves but not the response selection. At present, cumulative evidence supports the hypothesis that language affects visual perception, such as color perception (Maier & Rahman, 2018; Thierry et al., 2009; see Wolff & Holmes, 2011 for a review). Dominant explanations for this relationship between language and visual perception are a series of simulation theories, such as the indexical hypothesis (Glenberg & Kaschak, 2002) and embodied language processing (Gallese & Lakoff, 2007). According to such theories, the brain regions responsible for perceiving visual stimuli are activated by the visual imagery evoked by language. When the participants in Experiment 1 observed the associated “bouncing/streaming” emoticon, the concept of these stimuli was embodied, which means that the brain regions responsible for the streaming and bouncing percepts were recruited, resulting in biased perception.

A second explanation comes from the task-based expectation (Meteyard et al., 2007). The association between the arousing term and emoticon in Experiment 1 might produce a top-down modulation as a consequence of the two moving objects, causing priming at the premotion stage, which in turn interferes with the decision at the judgment stage, resulting in biased judgment or response.

Although the presented findings cannot be used to specify the mechanism underlying the arousal effect observed in Experiment 1 and Gobara et al. (2018), it is evident that the emotional processing per se does not solely modulate stream/bounce perception. Rather, emotional processing affects the stream/bounce judgment only in the context of the language processing. Interestingly, recent studies (Grove et al., 2012; Zeljko & Grove, 2017) using signal detection theory (Green & Swets, 1966; Macmillan & Creelman, 2005) in the stream/bounce paradigm demonstrated that the decisional criterion, rather than perceptual sensitivity, is responsible for at least the ABE (see also Grassi & Casco, 2012). This evidence supports our claim that the language-based response bias somehow contributes to the arousal effect on the stream/bounce judgment. Further investigations using signal detection theory in our paradigm are needed with regard to how emotional and language processing interact with each other in stream/bounce judgment. Also, we are interested in the degree to which language-based processing modulates the stream/bounce judgment in other contexts, such as ABE (Sekuler et al., 1997; Watanabe & Shimojo, 2001). For example, Sanabria et al. (2004) used an objective measure (i.e., the response time required to discriminate the target’s motion direction), instead of subjective reports from participants, of the effect of an auditory cue on the stream/bounce perception. They found that an auditory cue facilitated the bouncing percept (i.e., ABE) even in the absence of the response bias. Our study conceptually followed their study and found the less effect of an arousal stimulus on the stream/bounce judgment. These results illuminate that the language-based modulation of the stream/bounce judgment might be specific to the domain of emotional processing. This is likely because the emotional stimulus could be easily associated with linguistic codes due to its meaningfulness compared with the meaningless auditory tone; future investigations are needed regarding this issue. Furthermore, in addition to the stream/bounce percepts, the reversed motion percept arises in the stream/bounce display, when the two moving objects overlapped with one another and then changed immediately in their direction of motion (Kawachi, 2016). It would be interesting to investigate whether language-based processing modulates not only “bouncing” but also other linguistic codes, such as “reversing.”

Note that current evidence indicates only that the arousal effect (Gobara et al., 2018) on the stream/bounce judgment attenuated in response to the current experimental task that did not involve linguistic codes. In other words, a possibility that some participants might unexpectedly use linguistic codes while performing the task remains. Although such the possibility cannot explain the present findings, whether linguistic codes indeed are not used with the current task should be properly investigated, which strengthens our claim. Future research regarding this issue would provide a better understanding of the nature of the stream/bounce phenomenon.

In conclusion, the previous phenomenon (i.e., the arousal effect on the stream/bounce judgment) reported by Gobara et al. (2018) is replicable, but their previous interpretation is disputable.

## Data Availability

The data sets generated during and/or analyzed during this study are available in the Harvard Dataverse repository, https://doi:10.7910/DVN/SWQN4B.
